# Differential regulation of two histidine ammonia-lyase genes during *Xenopus* development implicates distinct functions during thyroid hormone-induced formation of adult stem cells

**DOI:** 10.1186/2045-3701-3-43

**Published:** 2013-11-13

**Authors:** Nga Luu, Luan Wen, Liezhen Fu, Kenta Fujimoto, Yun-Bo Shi, Guihong Sun

**Affiliations:** 1 Section on Molecular Morphogenesis, Program in Cellular Regulation and Metabolism (PCRM), Eunice Kennedy Shriver National Institute of Child Health and Human Development (NICHD), National Institutes of Health (NIH), 18 Library Dr., 20892 Bethesda, Maryland, USA; 2 School of Basic Medical Sciences, Wuhan University, 430072 Wuhan, P.R. China; 3 Present address: Division of Gene Structure and Function, Research Center for Genomic Medicine, Saitama Medical University, 1397-1 Yamane, 350-1241 Hidaka-shi, Saitama, Japan

**Keywords:** Thyroid hormone, Metamorphosis, *Xenopus*, Thyroid hormone receptor, Stem cells, Intestine, Histidase

## Abstract

**Background:**

Organ-specific, adult stem cells are essential for organ-homeostasis and tissue repair and regeneration. The formation of such stem cells during vertebrate development remains to be investigated. Frog metamorphosis offers an excellent opportunity to study the formation of adult stem cells as this process involves essentially the transformations of all larval tissues/organs into the adult form. Of particular interest is the remodeling of the intestine. Early studies in *Xenopus laevis* have shown that this process involves complete degeneration of the larval epithelium and de novo formation of adult stem cells through dedifferentiation of some larval epithelial cells. A major advantage of this metamorphosis model is its total dependence on thyroid hormone (T3). In an effort to identify genes that are important for stem cell development, we have previously carried out tissue-specific microarray analysis of intestinal gene expression during *Xenopus laevis* metamorphosis.

**Results:**

We report the detailed characterization of one of the genes thus identified, the histidine ammonia-lyase (HAL) gene, which encodes an enzyme known as histidase or histidinase. We show that there are two duplicated HAL genes, HAL1 and HAL2, in both *Xenopus laevis* and *Xenopus tropicalis*, a highly related but diploid species*.* Interestingly, only HAL2 is highly upregulated by T3 and appears to be specifically expressed in the adult intestinal progenitor/stem cells while HAL1 is not expressed in the intestine during metamorphosis. Furthermore, when analyzed in whole animals, HAL1 appears to be expressed only during embryogenesis but not metamorphosis while the opposite appears to be true for HAL2.

**Conclusions:**

Our results suggest that the duplicated HAL genes have distinct functions with HAL2 likely involved in the formation and/or proliferation of the adult stem cells during metamorphosis.

## Background

The adult mammalian intestine has long been used as a model organ to study adult organ-specific stem cells due to the rapid self-renewal of the intestinal epithelium throughout adult life [1-7]. This renewal of the epithelium is accomplished through constant proliferation of the stem cells located at the base of each intestinal crypt and subsequent differentiation as the cells move along the crypt-villus axis. After a finite period of time, the differentiated epithelial cells at the tip of the villus undergo apoptosis, completing the renewing cycle. Interestingly, this process is conserved across all vertebrates with the epithelium renewed every 1-6 days in mammals [1-3] and about 2 weeks in frogs [8].

Extensive studies on the mammalian intestine have revealed several signaling pathways important for intestinal embryogenesis and cell renewal and contributed to our understanding of intestinal homeostasis and neoplasia [3,9-11]. However, the molecular mechanisms regulating the formation of the adult stem cells during development are poorly understood. The development of the adult intestine in mammals takes place in two phases: 1) the formation of a primitive but functional intestine during embryogenesis and 2) subsequent maturation of the primitive intestine into the adult form around the neonatal period [3,7,9-11]. This second period is characterized by the presence of high levels of plasma thyroid hormone (T3). Importantly, several molecular and genetic studies have suggested that the formation of the mammalian adult intestine is dependent on T3 [10-16]. However, the difficulty to manipulate the mammalian embryos and neonates, as both are dependent on the mother has made it nearly impossible to study how T3 regulates the formation of adult intestinal stem cells.

Amphibian metamorphosis shares strong similarities with the postembryonic development in mammals, including intestinal maturation and the presence of high levels of T3 in the plasma [17,18]. Importantly, amphibian metamorphosis is totally dependent on T3 and can be easily manipulated by adding T3 to premetamorphic tadpoles to induce precocious metamorphosis or by blocking T3 function or synthesis to inhibit the process [17,18]. Furthermore, the adult intestine in amphibians, which is formed during metamorphosis, is similar to that in adult mammals. In the South African clawed frog *Xenopus laevis*, the larval intestine is a simple tubular structure consisting of a single layer of primary epithelium and thin layers of connective tissue and muscles. During metamorphosis, the larval epithelial cells undergo degeneration through programmed cell death or apoptosis. Concurrently, adult intestinal stem cells are formed de novo and eventually lead to the formation of a multi-folded adult epithelium surrounded by well-developed connective tissue and muscles [4,19]. In the frog, the adult epithelium is renewed along the trough-crest axis of the intestinal folds, similar to the mammalian crypt-villus axis. This process thus offers a unique opportunity to study how adult organ-specific stem cells are formed during vertebrate development [4,5,7].

Earlier studies have shown that the adult stem cells are formed through dedifferentiation of some cells in the larval intestinal epithelium [4,20-25]. More recent genetic and organ culture studies have shown that T3 can induce some cells within the larval epithelium to undergo autonomous dedifferentiation into precursor form of the adult intestinal stem cells [22,26]. However, such cells cannot develop into adult stem cells unless T3 signal is also present in the rest of the intestine or the non-epithelium (non-Ep), suggesting that T3 action in the non-Ep may function by helping to form the stem cell niche for the developing adult stem cells [20,22,26]. To identify the genes and signal processes induced by T3 in the epithelial and non-epithelial tissues during stem cell development, we have recently carried out a genome-wide microarray analysis on RNA isolated from the Ep and non-Ep of the intestine at different stages of metamorphosis [27]. Among the genes that were highly induced exclusively in Ep cells was the *Xenopus* homolog of the mammalian histidine ammonia-lyase or HAL.

HAL encodes a cytosolic enzyme known as histidase or histidinase. Histidase catalyzes the first reaction in histidine catabolism, the nonoxidative deamination of L-histidine to trans-urocanic acid and ammonia (http://www.ncbi.nlm.nih.gov/gene?Db=gene&Cmd=ShowDetailView&TermToSearch=3034) [28-31]. Histidase deficiency in human leads to histidinemia or histidinuria, a rare autosomal recessive metabolic disorder. Typical phenotypes associated with histidinemia include increased levels of plasma histidine, histamine, and imidazole, while decreased levels of the urocanic acid. Children with histidinemia may have hyperactivity, speech impediment, developmental delay, learning difficulties, and sometimes mental retardation (http://en.wikipedia.org/wiki/Histidinemia) [31-35]. The mechanisms underlying the histidase-deficiency mediated pathogenesis and what roles HAL plays during normal development remain to be investigated.

Here we demonstrate that there are two HAL genes in frogs due to a gene duplication event after the separation of amphibians from mammals. More importantly, we show that during metamorphosis, the two genes are regulated in a tissue- and gene-dependent manner in the intestine. In particular, HAL2 has no detectable expression before or after metamorphosis but its mRNA level was highly upregulated at the climax of metamorphosis. Spatially, HAL2 expression appeared to be specifically expressed in the newly formed adult progenitor/stem cells. In contrast, HAL1 expression is not detectable at any stages during intestinal metamorphosis. At the whole animal level, HAL1 but not HAL2 is expressed during embryogenesis while HAL2 but not HAL1 is expressed during metamorphosis. These results suggest distinct, tissue-specific roles for the two HAL genes during *Xenopus* development with HAL2 likely playing an important role in adult stem cell development and/or proliferation.

## Results

### The HAL gene was duplicated in *Xenopus*

**Figure 1:**
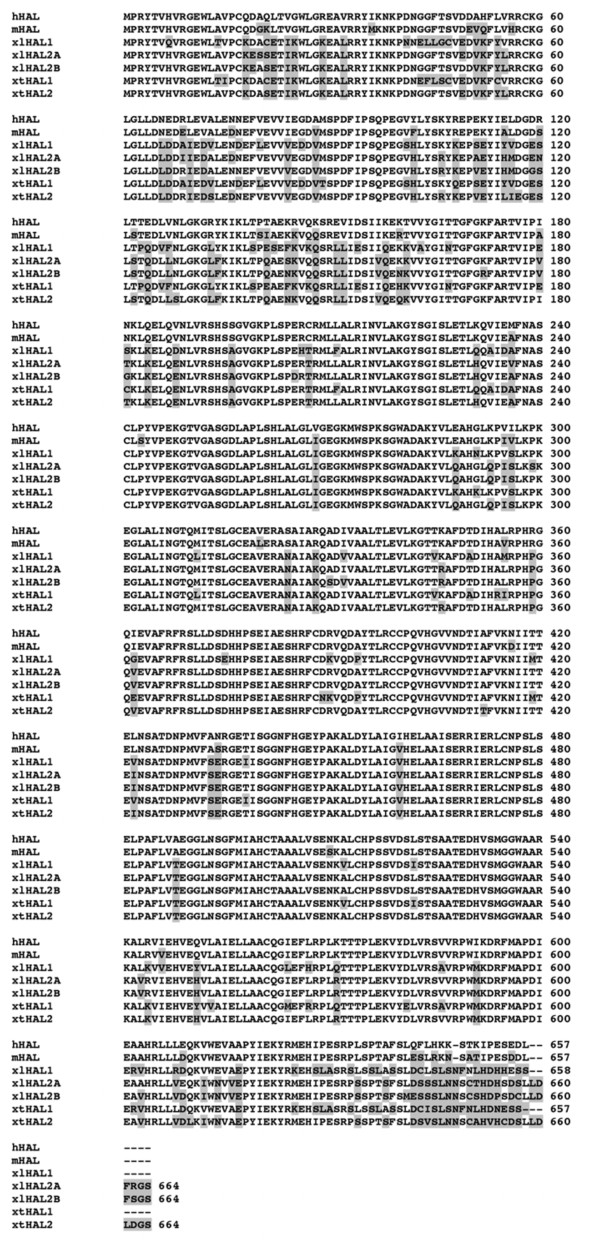
**The two *Xenopus *histidine ammonia lyase (HAL) genes encode proteins highly homologous to mammalian HAL.** Sequence alignment was carried out among the human (h), mouse (m) and the three HALs from *Xenopus laevis* (xl) and two HALs *Xenopus tropicalis* (xt), a diploid species. Note that the proteins are highly homologous throughout the entire length. The HAL1 and HAL2 from *Xenopus laevis* and *Xenopus tropicalis* shared about 84% identity between each other, similar to their homologies to the mammalian HAL.

At the climax of metamorphosis in *Xenopus laevis*, the larval intestinal epithelial cells undergo apoptosis and adult epithelial progenitor/stem cells are formed and rapidly proliferate [8,19,36]. Earlier studies indicate that around stages 61/62, a large fraction of the cells in the intestinal epithelium are the adult progenitor/stem cells [8,36]. Thus, genes expressed in the intestinal epithelium at the climax of metamorphosis are likely to be important for the progenitor/stem cells. Our earlier tissue-specific microarray analysis on the *Xenopus laevis* intestine revealed that the expression of mRNAs corresponding to a microarray cDNA probe (Probe ID# A_10_P010027, GenBank accession No. BE507589) homologous to the mammalian HAL gene was strongly upregulated specifically in the epithelium at the climax of metamorphosis [27]. Searches of GenBank sequence database revealed that that in *Xenopus laevis,* there were three distinct cDNA sequences (designated xlHAL2A, xlHAL2B, and xlHAL1) identical or highly homologous to the microarray probe. One of them, HAL2A, corresponded to the probe on the microarray and another HAL2B, was 94% and 96% identical to HAL2A at the cDNA and amino acid sequence level, respectively (Table 1), suggesting that HAL2A and B represent two duplicated copies of the HAL2 genes in the pseudotetraploid *Xenopus laevis* genome. The third one, HAL1, shared 81% and 84% identities with *Xenopus laevis* HAL2 genes at the cDNA and amino acid sequence level, respectively (Table 1), much lower than those between the two HAL2 genes. In addition, both HAL1 and HAL2 were highly homologous (about 80% identity) to the mouse or human HAL, which is encoded by a single gene (Figure 1).

**Table 1:** % identity from pairwise sequence alignment among HALs from different species

	**hHAL**	**mHAL**	**xlHAL1**	**xlHAL2A**	**xlHAL2B**	**xtHAL1**	**xtHAL2**
**hHAL**		***87.5***	***72.4***	***74.2***	***74.4***	***72.4***	***74***
**mHAL**	93.9		***72.3***	***73.4***	***73.8***	***72.6***	***73.5***
**xlHAL1**	80.1	79.5		***80.9***	***80.6***	***92.5***	***81.0***
**xlHAL2A**	84.8	85.1	83.9		***93.9***	***81.1***	***93.5***
**xlHAL2B**	84.3	84.3	83.6	96.4		***81.5***	***92.8***
**xtHAL1**	80.4	79.6	95.4	83.6	83.4		***82.0***
**xtHAL2**	84.3	84.5	84.5	95.8	95.3	84.5	

(Amino acid sequence: lower left half; and coding region nucleotide sequence: upper right half and in bold italics).

**Figure 2:**
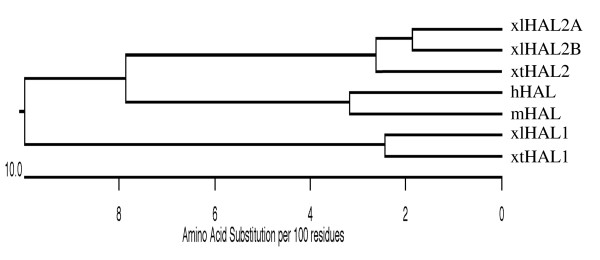
**Phylogenetic tree of HALs from different species as shown in Figure**1**suggests that the two HAL genes were duplicated after the separation of mammals and amphibians but prior to the separation of *Xenopus laevis *** **and *Xenopus tropicalis *** **or that mammals lost the HAL1 gene during evolution.**

The existence of at least three different HAL genes in the pseudotetraploid *Xenopus laevis* suggests that there may be two different HAL genes even in diploid amphibians. To investigate this possibility, we took advantage of the genome sequence information for the diploid frog species, *Xenopus tropicalis*, which is highly related to *Xenopus laevis*, including conservations at the molecular level during metamorphosis [37-44]. Analysis of the genomic sequence data revealed the presence of two HAL genes in *Xenopus tropicalis*. The predicted amino acid sequences of the two HAL genes were highly conserved between the two *Xenopus* species, with the corresponding HAL genes in the two species sharing about 93% and 95% identity at the cDNA and amino acid sequence levels, respectively (Table 1 and Figure 1), while the two HALs in either *Xenopus* species were only 82% and 84% identical to each other at the cDNA and amino acid sequence levels, respectively (Table 1 and Figure 1). Phylogenetic analysis of the *Xenopus* and mammalian HALs showed distinct clustering of the HAL1 and HAL2 genes and that the mammalian HAL gene was more closely related to HAL2. These results suggest that either one of the HAL genes was lost in mammals or that there was a HAL gene duplication event in *Xenopus* during evolution after the separation of mammals and amphibians but prior to the separation of *Xenopus laevis* and *Xenopus tropicalis* (Figure 2).

### Distinct tissue-specific regulation of the two HAL genes during intestinal metamorphosis in *Xenopus laevis*

**Figure 3:**
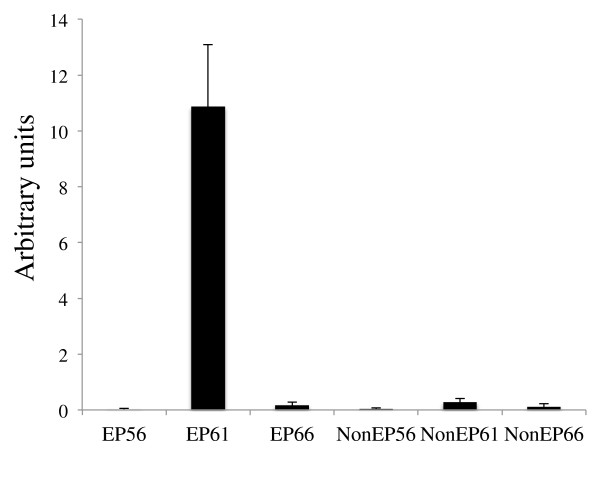
**Upregulation of HAL2 gene only in the epithelium during *Xenopus laevis *intestinal metamorphosis.** The epithelium (Ep) and the rest of the intestine (non-Ep), which is made of mainly the connective tissue, were isolated from tadpoles at stage 56 (the onset of metamorphosis, also referred to as a premetamorphic stage), stage 61 (the climax of metamorphosis), and stage 66 (the end of metamorphosis) [27]. The total RNA from the Ep and non-Ep was used for qRT-PCR analysis of HAL2 gene expression. Note that HAL2 was exclusively expressed in the Ep at the climax of metamorphosis.

Sequence comparison showed that the original cDNA on the microarray corresponded to the HAL2A gene. The microarray data indicated that HAL2 was specifically upregulated and exclusively expressed in the epithelium at the climax of metamorphosis [27]. To confirm this and to determine whether HAL1 was similarly regulated, we made use of the RNA isolated from enzymatically separated intestinal epithelium (Ep) and the non-Ep (the rest of the intestine) at different stages of metamorphosis [27]. Quantitative (q) RT-PCR with gene specific primers showed that as expected, HAL2 had no detectable expression in the Ep of premetamorphic (stage 56) or postmetamorphic (stage 66) animal intestine but was highly expressed in the Ep at the climax (stage 61) (Figure 3) (note that due to the high degree of homology between HAL2A and B, it is difficult to determine the expression of the individual HAL2 genes. All the data here represented the sum of both HAL2 genes). No expression was found in the non-Ep at any stages (Figure 3), consistent with the microarray data [27].

Unlike HAL2, we were not able to detect any HAL1 expression by RT-PCR (data not shown). Thus, the two HAL genes were differentially regulated during metamorphosis with HAL2 exclusively expressed in the intestinal Ep at the climax of metamorphosis while HAL1 expression was absent in the intestine throughout metamorphosis.

**Figure 4:**
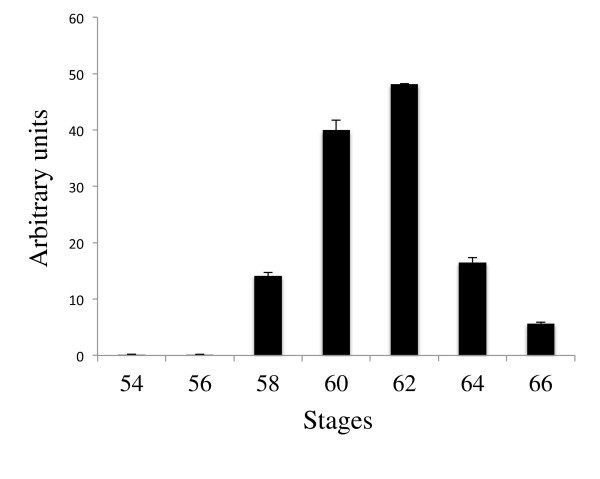
**Upregulation of HAL2 during intestinal metamorphosis.** Total RNA isolated from the intestine of animals from premetamorphic stage 54 to the end of metamorphosis (stage 66) was subjected to qRT-PCR analysis for HAL2 gene expression. Note that again HAL2 was found to have little or no expression before or after metamorphosis but was drastically upregulated during metamorphosis.

During metamorphosis, the tissue composition changes in the intestine. In premetamorphic tadpoles, the epithelium predominates while the connective tissue and muscles exist as very thin layers [8,36,45,46]. The connective tissue and muscles subsequently develop extensively with the amounts of these two tissues relative to the epithelium reaching much higher levels after metamorphosis. To determine whether the total intestinal expression of the HAL genes changes during metamorphosis and to rule out any potential effects on mRNA levels by the enzymatic separation of the Ep and non-Ep, total RNA was isolated from the small intestine of animals from premetamorphic stage 54 to the end of metamorphosis (stage 66) and subjected to qRT-PCR analysis. As shown in Figure 4, HAL2 had little or no expression in the intestine before or after metamorphosis but was drastically upregulated during metamorphosis, while HAL1 had no detectable expression (not shown), consistent with the expression data from isolated intestinal tissues (Figure 3).

### Regulation of HAL2 genes by T3

**Figure 5:**
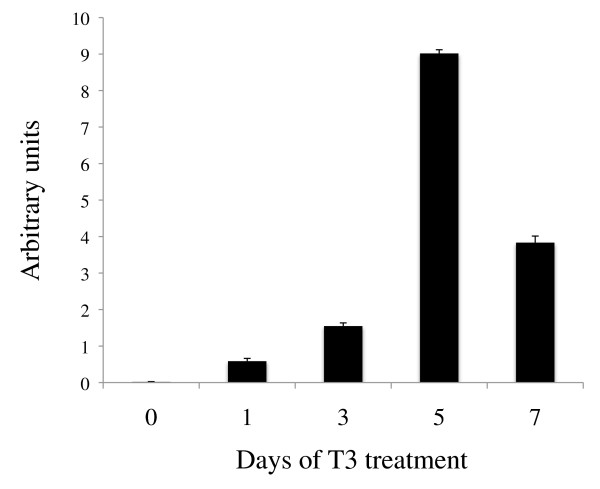
**Dramatic upregulation of HAL2 by T3-treatment of premetamorphic tadpoles.** Stage 54 premetamorphic tadpoles were treated with 10 nM T3 for 0-7 days and total intestinal RNA was isolated for expression analysis. Note that HAL2 expression was upregulated significantly after 1 day of treatment and the expression continued to increase dramatically during the treatment, peaking after 5 days.

T3 is the causative agent of metamorphosis and T3 treatment of premetamorphic tadpoles can induce precocious metamorphosis including intestinal remodeling. The tissue-specific expression patterns of the HAL genes suggest that HAL2 is likely regulated by T3 either directly or indirectly. To investigate this, premetamorphic tadpoles at stage 54 were treated for 0-7 days with 10 nM T3, a concentration close to the physiological plasma T3 level at the climax of metamorphosis [47]. Total RNA was isolated from the intestine and subjected to qRT-PCR analysis for HAL2 expression. Consistent with the developmental expression patterns, HAL2 upregulation was observed as early as only 1 day of T3 treatment, with expression levels peaked after 5 days of treatment (Figure 5).

### HAL2 appears to be highly expressed specifically in the adult progenitor/stem cells during intestinal metamorphosis

**Figure 6:**
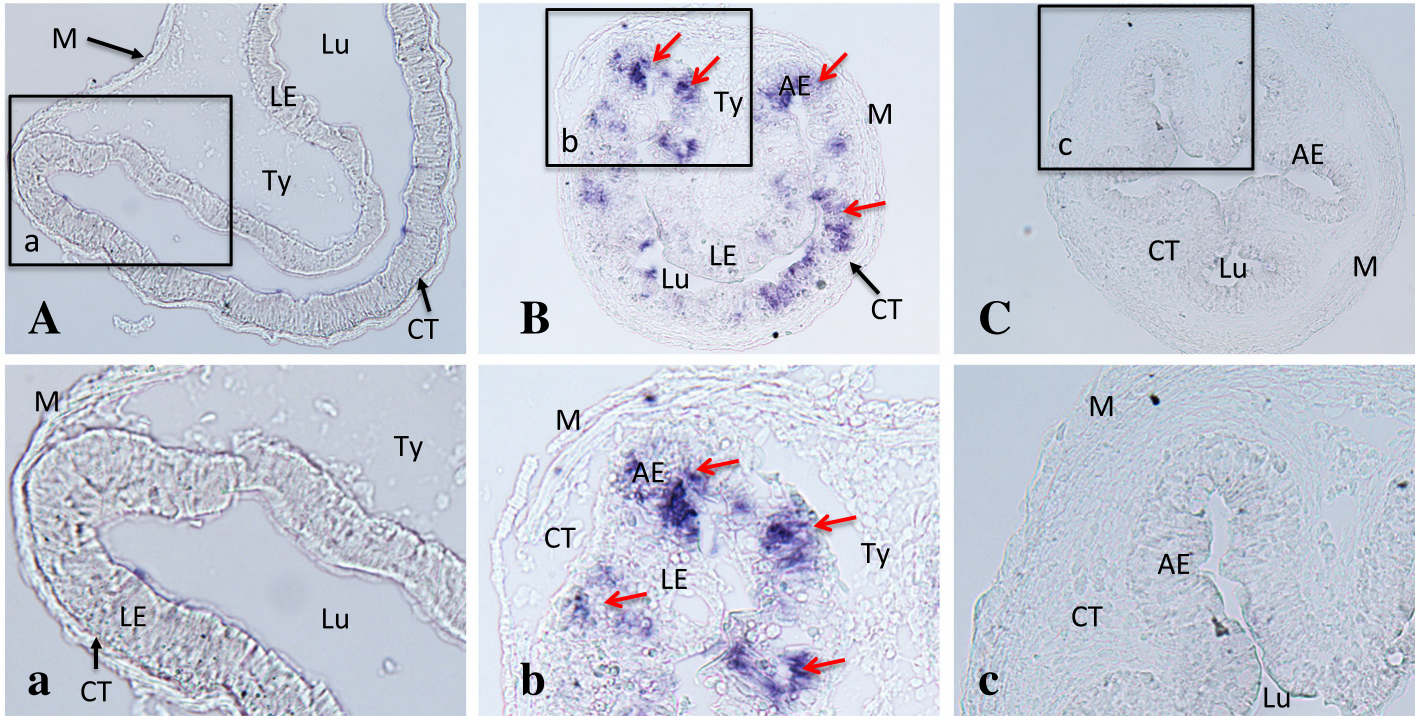
***In situ *hybridization analysis suggests specific expression of HAL2 in the proliferating adult intestinal progenitor/stem cells.** ISH on intestinal cross-sections at stages 54 **(A)**, 61 **(B)**, and 66 **(C)** was carried out with anti-sense probe for HAL2. An enlarged photo of the boxed areas **(a, b, c)** in the top panels are shown in the lower panels. Note that HAL2 staining was found to be in clusters of cells located in between the dying larval epithelial cells and connective tissue at the climax of metamorphosis, where the proliferating adult epithelial cells are located [8,19,36,48], consistent with the expression data in Figures 3 and 4. AE, adult epithelium; CT, connective tissue; Lu, lumen; LE, larval epithelium; M, muscle; Ty, typhlosole.

The exclusive expression of HAL2 in the Ep at the climax of intestinal metamorphosis suggests that it was expressed in the developing adult progenitor/stem cells. To localize the expression HAL1 and HAL2 in the intestine, we carried out *in situ* hybridization analyses by using cRNA probes derived from a less conserved coding region of the two HAL genes that were less conserved. The HAL1 and HAL2 specific probes consisted of 669 nucleotides and share 77% homology. As shown in Figure 6, the HAL2 probe detected strong signals exclusively in clusters of cells in the Ep at the climax of metamorphosis but not in pre- or post-metamorphic animals (Figure 6), consistent with the qRT-PCR findings (Figures 3 and 4). Earlier studies have shown that these Ep cell clusters are adult progenitor/stem cells, while at this stage, the remaining larval epithelial cells are undergoing apoptosis [8,19,36,48]. The dying larval epithelial cells did not express HAL2 (Figure 6). Unlike HAL2, the HAL1 in situ probe failed to detect any signal in the intestine throughout metamorphosis, consistent with the RT-PCR results (data not shown). These findings suggest that HAL2 expression was specific to the adult progenitor/stem cells while HAL1 was not expressed in the remodeling intestine.

### HAL1 and HAL2 have distinct temporal expression profiles during *Xenopus* development

**Figure 7:**
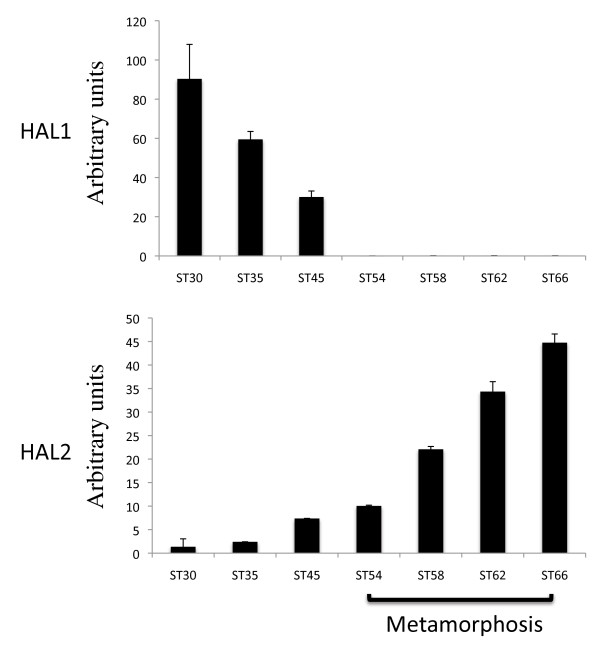
**Distinct expression profiles of HAL1 and HAL2 during development.** Total RNA isolated from whole animals from embryonic stage 30 to the end of metamorphosis (stage 66) was subjected to quantitative RT-PCR analysis for HAL1 and HAL2 gene expression. Note that HAL1 was expressed strongly in embryos and repressed after stage 45 when tadpole feeding begins while HAL2 was upregulated by stage 45 and its expression continued to rise till the end of metamorphosis.

The presence of HAL1 cDNA sequence in the GenBank database suggests that HAL1 is expressed in some tissues and/or at some stages of development. To investigate this possibility, we isolated total RNA from whole animals from embryonic stage 30 to the end of metamorphosis (stage 66) and carried out RT-PCR analysis by using the same primer sets as used above for their expression in the intestine. The results showed that HAL1 was highly expressed in embryos at stag 30 but was repressed after stage 45 when tadpole feeding begins (Figure 7). In contrast, HAL2 mRNA was not detected at the stages analyzed during embryonic development but was upregulated as tadpole feeding begins and its expression continues to rise during metamorphosis to reach high levels in postmetamorphic frogs. Thus, the two HAL genes may have complementary roles during *Xenopus* development.

## Discussion/conclusions

Intestinal remodeling during amphibian metamorphosis offers an excellent opportunity to study the development of adult organ-specific stem cells in vertebrates due to the de novo formation of the adult stem cells during this process and its resemblance to the maturation of adult intestine during postembryonic development in mammals [4,5]. Making use of its total dependence on T3, we have previously identified many candidate stem cell genes in the Ep and non-Ep, which contributes to the formation of the stem cell niche [26,49]. Our analysis of one of such Ep genes, revealed the existence of two HAL genes in frogs. More importantly, the distinct expression patterns suggest that the two HAL genes have different roles during *Xenopus* development.

Our sequence analyses revealed that the HAL gene was duplicated in amphibians as two genes are present in the diploid *Xenopus tropicalis*. Importantly, the two genes had distinct temporal-spatial expression patterns during development in *Xenopus laevis*. In whole animals, HAL1 expression was high during embryonic development and was repressed after stage 45 when embryogenesis is complete and tadpole feeding begins. Subsequently, HAL1 expression was absent in tadpoles throughout the rest of the development. On the other hand, HAL2 was not expressed during embryogenesis and its mRNA levels were upregulated as tadpole feeding begins at stage 45. Its expression increased further during metamorphosis to reach high levels at the end of metamorphosis. Thus, the two HAL genes appear to play complementary roles during *Xenopus* development. HAL1 appears to be involved in embryogenesis while HAL2 participates in metamorphosis. Interestingly, while in the intestine, HAL2 is only expressed during the period of stem cell development and proliferation but not in the adult intestine, HAL2 expression is high in postmetamorphic frog. Thus, HAL2 is also important for the function/homeostasis of one or more adult organs such as liver, which would not be surprising given the importance of histidine metabolism in adult physiology [28,31].

The distinct temporal profiles of the two HAL genes in whole animals suggest that HAL2 but not HAL1 is important for metamorphosis. During metamorphosis, the larval intestinal epithelial cells undergo apoptosis with a very small fractions of the epithelial cells undergo dedifferentiation to form the adult progenitor/stem cells [4,5,26]. At the climax of metamorphosis (stage 62), essentially all the cells in the epithelium are the proliferating adult progenitor/stem cells with only a small fraction of remaining larval cells that are undergoing apoptosis [8,36]. Interestingly, HAL2 was only expressed at the climax of metamorphosis in the adult progenitor/stem cells but not in the differentiated larval or adult epithelial cells. In addition, T3 treatment of premetamorphic tadpoles induced HAL2 expression promptly and dramatically, suggesting that HAL2 may be regulated by T3 directly at the transcription levels via thyroid hormone receptor binding to its promoter region [50]. In contrast, HAL1 was not upregulated by T3 treatment, consistent with its lack of expression during intestinal metamorphosis. Thus, the two duplicated HAL genes are differentially regulated by T3 during *Xenopus* development. In this regard, it is interesting to note that the matrix metalloproteinase gene MMP9 is a single gene in mammals but duplicated in amphibians with one of the duplicated genes (MMP-9 TH) expressed in the regressing tail during metamorphosis, while the other (MMP-9) expressed in embryos [51]. Thus, many duplicated genes in amphibians may have evolved to serve distinct roles during development.

If and how HAL2 participates in stem cell formation/proliferation remains to be investigated. HAL encodes an enzyme known as histidase [28-31]. At biochemical level, histidase catalyzes the first step in the histidine catabolism pathway, i.e., the deamination of histidine to ammonia and urocanic acid. This raises a few potentially testable hypotheses: the catalysis of histidine and/or its product urocanic acid is important for stem cell development and/or proliferation. In this regard, it is worth noting that Wang et al. [52] reported that mouse embryonic stem cells are dependent on threonine catabolism. These authors discovered high levels of expression of threonine dehydrogenase gene in cultured embryonic stem (ES) cells [52]. By using culture media individually deprived of each of the 20 amino acids, they showed that the ES cells are dependent on threonine. As threonine dehydrogenase catalyzes the initial, rate-limiting step in a threonine catabolism pathway, their findings suggest that ES cells might exist in a metabolic state that facilitates rapid growth, in part by using threonine catabolism to generate glycine and acetyl-coenzyme A (acetyl-CoA), with glycine facilitating one-carbon metabolism and acetyl-CoA feeding the tricarboxylic acid cycle [52]. It is thus tempting to speculate that HAL2 may also be critical for the rapid proliferating of the adult intestinal stem cells during metamorphosis by initiating histidine catabolism.

Histidase is encoded by a single HAL gene in mammals. Its deficiency has been implicated to be the underlying cause for histidinemia in human and mouse [31-35], which in human can lead to abnormal development in children, including mental retardation (http://en.wikipedia.org/wiki/Histidinemia) [31,32,34,35]. How histidase deficiency leads to such developmental abnormalities remains to be investigated. Our discovery that one of the two duplicated HAL genes in *Xenopus* likely play a crucial role in adult intestinal stem cell development suggests that HAL may affect human development by influencing the maturation of adult organs, such as the brain. Our findings further offer a possible model to investigate how HAL functions during organ maturations, which are critical to neonatal development in children, a period likely most sensitive to histidinemia.

### Experimental animals

*Xenopus laevis* tadpoles were purchased from NASCO or produced in the laboratory. Developmental stages were determined as described [53]. When indicated, stage 54 tadpoles were treated with 10 nM T3 for 0-7 days at 18°C. All animals were maintained and used as approved by the Animal Use and Care Committee of Eunice Kennedy Shriver National Institute of Child Health and Human Development, National Institutes of Health.

### Tissue collection and RNA isolation

Total RNA isolation from whole animals was described before [54,55]. The small intestine was isolated from staged tadpoles with or without T3 treatment and flushed of contents before the isolation of total intestinal RNA. Total RNA was extracted with TRIZOL Reagent (Invitrogen) from the isolated intestines. Total RNA from the intestinal epithelium (Ep) and the rest of the intestine or non-Ep was isolated as described before [27,56]. Total RNA was made DNA-free with DNase I treatment and recovered with Phenol: Choloform: Isoamyl alcohol (25:24:1) extraction and isopropanol precipitation.

### Sequence comparison

HAL nucleotide and amino acid sequences were retrieved from GenBank database (http://www.ncbi.nlm.nih.gov). The accession numbers were: NM_002108 (nucleotide) and NP_002099 (protein) for human HAL (hHAL); NM_010401 (nucleotide) and NP_034531 (protein) for mouse HAL (mHAL); NM_001093175 (nucleotide) and NP_001086644 (protein) for *Xenopus laevis* HAL1 (xlHAL1); NM_001127093 (nucleotide) and NP_001120565 (protein) for *Xenopus tropicalis* HAL1 (xtHAL1); NM_001105266 (nucleotide) and NP_001098736 (protein) for *Xenopus laevis* HAL2A (xlHAL2A); NM_001092595 (nucleotide) and NP_001086064 (protein) for *Xenopus laevis* HAL2B (xlHAL2B); and NM_001006831 (nucleotide) and NP_001006832 (protein) for *Xenopus tropicalis* HAL2 (xtHAL2), respectively. Sequence alignment and phylogenetic analysis were done with DNASTAR (DNASTAR, WI).

### qRT-PCR

This was done as described [16,57] by using gene-specific primers and SYBR® Green I dye (Applied Biosystems) with an ABI Prism 7000 sequence detection system (Applied Biosystems). The expression levels of HAL genes were normalized to that of the control gene, the somatic elongation factor 1 alpha (EF-1α) [41]. The primer sequences were 5′- CTATCCACCGCCAAACATCT-3′ and 5′- CCATCTCAGCAGCTTCCTTC-3′ for EF1α, 5′-AGCTGCTCACAGGTTGCTAGTT-3′ and 5′-AAGAGTCCAATGAAAAAGATGTA-3′ for HAL2 (the primers amplify both HAL2A and 2B), and 5′-ATGCAATCGCTAAACAGGCTGAC-3′ and 5′-GTCCTGAACTTTATCACAAAATCG-3′ for HAL1. The experiments were repeated at least 2-3 times with 3-5 animals/sample and similar conclusions.

### In situ hybridization (ISH)

A 669 bp fragment in the coding region of HAL1 and HAL2 cDNA was PCR amplified using the following primers: HAL1 forward 5′ACTGTTCCATGTAAAGATGCTTGTG 3′, HAL1 reverse 5′ AGCATCAATAGCTTGTTGTAACG 3′; HAL2 forward 5′ GCCGTGCCTTGCAAAGAATCTTC 3′, HAL2 reverse 5′ CACTTCAATGACTTGATGCAATG 3′. The PCR product was subcloned into pCR-Blunt II-TOPO cloning vector (Invitrogen). The resulting plasmid was linearized to synthesize sense and antisense probes either with SP6 or T7 RNA polymerase, respectively, by using a digoxigenin (DIG) RNA Labeling kit (Roche Applied Science, Indianapolis, IN, USA). Intestinal fragments were isolated from the anterior part of the small intestine from tadpoles at indicated stages, fixed in 4% MEMFA followed by cryo-sectioning. Tissue sections cut at 10 μm were subjected to ISH by using sense or antisense probe as previously described [58]. Photographs were taken by using a digital CCD color camera (Retiga Exi, QImaging) attached to an optical microscope (BX60, Olympus).

## Competing interests

The authors declare that they have no competing interests.

## Authors’ contributions

NL, LW, LF, KF, and GS designed and carried out experiments and interpreted the findings. NL, LF, YBS, and GS prepared the manuscript. All authors read and approved the final manuscript.
